# Supplementary material for: Predicting lymph node metastasis from primary tumor histology and clinicopathologic factors in colorectal cancer using deep learning
Source: Commun Med (Lond). 2023 Apr 24;3:59. doi: 10.1038/s43856-023-00282-0 (PMC10125969; doi:10.1038/s43856-023-00282-0)
Supplement: Supplementary file 1 — Reporting Summary [file 43856_2023_282_MOESM1_ESM.pdf]

nature research

Corresponding author(s): Justin Krogue, MD

Last updated by author(s): 3/24/2023

Reporting Summary

Nature Research wishes to improve the reproducibility of the work that we publish. This form provides structure for consistency and transparency in reporting. For further information on Nature Research policies, see our Editorial Policies and the Editorial Policy Checklist.

Statistics

For all statistical analyses, confirm that the following items are present in the figure legend, table legend, main text, or Methods section.

n/a	Confirmed	
☐	☒	The exact sample size (*n*) for each experimental group/condition, given as a discrete number and unit of measurement
☐	☒	A statement on whether measurements were taken from distinct samples or whether the same sample was measured repeatedly
☐	☒	The statistical test(s) used AND whether they are one- or two-sided<br>*Only common tests should be described solely by name; describe more complex techniques in the Methods section.*
☐	☒	A description of all covariates tested
☐	☒	A description of any assumptions or corrections, such as tests of normality and adjustment for multiple comparisons
☐	☒	A full description of the statistical parameters including central tendency (e.g. means) or other basic estimates (e.g. regression coefficient) AND variation (e.g. standard deviation) or associated estimates of uncertainty (e.g. confidence intervals)
☐	☒	For null hypothesis testing, the test statistic (e.g. *F*, *t*, *r*) with confidence intervals, effect sizes, degrees of freedom and *P* value noted<br>*Give P values as exact values whenever suitable.*
☒	☐	For Bayesian analysis, information on the choice of priors and Markov chain Monte Carlo settings
☐	☒	For hierarchical and complex designs, identification of the appropriate level for tests and full reporting of outcomes
☐	☒	Estimates of effect sizes (e.g. Cohen's *d*, Pearson's *r*), indicating how they were calculated

Our web collection on statistics for biologists contains articles on many of the points above.

Software and code

Policy information about availability of computer code

Data collection	n/a
Data analysis	The image-similarity deep learning model was developed using Tensorflow (2.10.0) deep learning framework. Clustering was performed using sklearn.cluster.KMeans from the scikit-learn (0.24.1) python package. Logistic regression modeling was done using statsmodels.discrete.discrete_model.Logit from the statsmodels (v0.12.2) python package.<br><br>In this work we use pre-trained deep learning models from 3 different approaches (Graph-RISE, BiT, SimCLR) to produce embeddings, and show that machine-features derived from these embedding achieve similar performance in LNM prediction. The BiT model has been open sourced and is available on TFHub (https://tfhub.dev/google/bit/s-r50x1/1). Code for generating and evaluating the machine-learned features while controlling for baseline features will be made available on GitHub (https://github.com/Google-Health/google-health/tree/master). Code for pretraining a SimCLR model is available at (https://github.com/google-research/simclr).

For manuscripts utilizing custom algorithms or software that are central to the research but not yet described in published literature, software must be made available to editors and reviewers. We strongly encourage code deposition in a community repository (e.g. GitHub). See the Nature Research guidelines for submitting code & software for further information.

Data

Policy information about availability of data

All manuscripts must include a data availability statement. This statement should provide the following information, where applicable:

- Accession codes, unique identifiers, or web links for publicly available datasets
- A list of figures that have associated raw data
- A description of any restrictions on data availability

This study utilized archived anonymized pathology slides, clinicopathologic variables, and outcomes from the Institute of Pathology and the Biobank at the Medical University of Graz and Stanford University. Interested researchers should contact K.Z. to inquire about access to Biobank Graz data and J.S. to inquire about access to Stanford University data; reasonable requests for research use will be considered and require ethics review prior to access.

Field-specific reporting

Please select the one below that is the best fit for your research. If you are not sure, read the appropriate sections before making your selection.

☒ Life sciences ☐ Behavioural & social sciences ☐ Ecological, evolutionary & environmental sciences

For a reference copy of the document with all sections, see nature.com/documents/nr-reporting-summary-flat.pdf

Life sciences study design

All studies must disclose on these points even when the disclosure is negative.

Sample size	As we are building a deep learning model, we simply used all data available to us retrospectively that met inclusion/exclusion criteria from our two data institutions.
Data exclusions	Exclusion criteria include:<br>- Incomplete clinical metadata (TNM staging, age, sex, tumor grade)<br>- Stage I and IV cases (as our primary dataset only had these)<br>- T1 and T2 cases (since we only had Stage II/III cases there is a confounding association between T-stage and lymph node metastasis. I.e., T1 and T2 cases are only included if they are lymph node positive)
Replication	We tested our model on an external dataset (Stanford cohort) to ensure it generalized. We have also open sourced our code so that our results may be replicated.
Randomization	Cases were lymph node positive or negative as per clinical diagnosis.
Blinding	No blinding was performed.

Reporting for specific materials, systems and methods

We require information from authors about some types of materials, experimental systems and methods used in many studies. Here, indicate whether each material, system or method listed is relevant to your study. If you are not sure if a list item applies to your research, read the appropriate section before selecting a response.

Materials & experimental systems

n/a	Involved in the study	
☒	☐	Antibodies
☒	☐	Eukaryotic cell lines
☒	☐	Palaeontology and archaeology
☒	☐	Animals and other organisms
☐	☒	Human research participants
☒	☐	Clinical data
☒	☐	Dual use research of concern

Methods

n/a	Involved in the study	
☒	☐	ChIP-seq
☒	☐	Flow cytometry
☒	☐	MRI-based neuroimaging

Human research participants

Policy information about studies involving human research participants

Population characteristics	This retrospective study utilized de-identified, digitized histopathology slides of primary colorectal samples and clinicopathologic metadata from colorectal cancer cases from the BioBank at the Medical University of Graz (MUG)17 and from Stanford University (SU). Slides were scanned using a Leica Aperio AT2 scanner at 20X magnification (0.5 µm/pixel). Institutional Review Board approval for this study was obtained from MUG (Protocol no. 30-184 ex 17/18) and SU (Protocol

no. 46762). Clinicopathologic metadata including pathologic TNM staging, age, sex, and tumor grade were extracted from de-identified clinical and pathology reports. When indicated in the report, presence of lymphatic invasion and venous invasion were also extracted. Only cases with complete clinicopathologic metadata for TNM staging, age, sex, tumor grade were included in this study. Patient characteristics of these cohorts are reported in Table 1.

Cases from MUG comprise archived stage II and stage III colorectal cases from 1984 to 2013. Cases from 1984-2007 were used for model development and feature selection (divided into training and tune sets) and cases from 2008-2013 were used as a temporal validation set. In the event of multiple cases for a given patient, only the primary resection was included. Cases from SU comprise all available archived stage II and III colorectal cancer cases and a random sample of available stage I and IV cases from 2007-2018 (one case per patient). The SU cases were used for external validation.

Development cohort (MUG):
Num. cases
2,921
Num. slides
21,260
Years
1984-2007
Pathologic Stage
Stage I
0 (0%)
Stage II
1,504 (51%)
Stage III
1,417 (49%)
Stage IV
0 (0%)
T-Category
T2
0 (0%)
T3
2,527 (87%)
T4
394 (13%)
N-Category
N0
1,504 (51%)
N1
792 (27%)
N2
438 (15%)
N3
187 (6%)
Age
<60
609 (21%)
60-69
850 (29%)
70-79
1,004 (34%)
>80
458 (16%)
Sex

Female
1,539 (53%)
Male
1,382 (47%)
Tumor Grade
Grade 1
113 (4%)
Grade 2
2,160 (74%)
Grade 3
648 (22%)
Lymphovascular Invasion
absent
2,499 (86%)
present
422 (14%)
Venous Invasion
absent
2,747 (94%)
present
174 (6%)

Temporal validation cohort (MUG):
Num. cases
670
Num. slides
6,440
Years
2008-2013
Pathologic Stage
Stage I
0 (0%)
Stage II
316 (47%)
Stage III
354 (53%)
Stage IV
0 (0%)
T-Category
T2
0 (0%)
T3
503 (75%)
T4
167 (25%)
N-Category
N0
316 (47%)
N1
201 (30%)
N2
147 (22%)
N3
6 (1%)
Age
<60
115 (17%)
60-69
165 (25%)
70-79
214 (32%)
>80
176 (26%)
Sex

Female
367 (55%)
Male
303 (45%)
Tumor Grade
Grade 1
36 (5%)
Grade 2
414 (62%)
Grade 3
220 (33%)
Lymphovascular Invasion
absent
499 (74%)
present
171 (26%)
Venous Invasion
absent
551 (82%)
present
119 (18%)

External validation cohort (SU)
Num. cases
550
Num. slides
5,373
Years
2007-2018
Pathologic Stage
Stage I

0 (0%)
Stage II
259 (47%)
Stage III
291 (53%)
Stage IV
0 (0%)
T-Category
T2
0 (0%)
T3
462 (84%)
T4
88 (16%)
N-Category
N0
259 (47%)
N1
188 (34%)
N2
103 (19%)
N3
0 (0%)
Age
<60
243 (44%)
60-69
108 (20%)
70-79
108 (20%)
>80
91 (17%)
Sex

Female
310 (56%)
Male
240 (44%)
Tumor Grade
Grade 1
177 (32%)
Grade 2
308 (56%)
Grade 3
65 (12%)
Lymphovascular Invasion
absent
379 (69%)
present
171 (31%)
Venous Invasion
absent
477 (87%)
present
73 (13%)

Recruitment	All patients present contained in the patholological archives from the two institutions who met inclusion/exclusion criteria were included
Ethics oversight	Institutional Review Board approval for this study was obtained from Medical University of Graz (Protocol no. 30-184 ex 17/18) and Stanford University (Protocol no. 46762)

Note that full information on the approval of the study protocol must also be provided in the manuscript.
