# Supplementary material for: Predicting lymph node metastasis from primary tumor histology and clinicopathologic factors in colorectal cancer using deep learning
Source: Commun Med (Lond). 2023 Apr 24;3:59. doi: 10.1038/s43856-023-00282-0 (PMC10125969; doi:10.1038/s43856-023-00282-0)
Supplement: Supplementary file 2 — Supplementary Material [file 43856_2023_282_MOESM2_ESM.pdf]

Predicting lymph node metastasis from primary tumor histology and clinicopathologic factors in colorectal cancer using deep learning

Authors:
Justin D. Krogue[1*], Shekoofeh Azizi[2], Fraser Tan[1], Isabelle Flament-Auvigne[3], Trissia Brown[3], Markus Plass[4], Robert Reihs[4], Heimo Müller[4], Kurt Zatloukal[4], Pema Richeson[5], Greg S. Corrado[1], Lily H. Peng[1], Craig H. Mermel[1,], Yun Liu[1], Po-Hsuan Cameron Chen[1], Saurabh Gombar[5], Thomas Montine[5], Jeanne Shen[5], David F. Steiner[1,†], Ellery Wulczyn[1,†]

Affiliations:
[1]Google Health, Palo Alto, California, United States of America
[2]Google Research, Brain Team, Toronto, Ontario, Canada
[3]Work done at Google Health via Vituity, Emeryville, CA, United States of America
[4]Medical University of Graz, Graz, Austria
[5]Department of Pathology, Stanford University School of Medicine, Stanford, California, United States of America
[†]Equal contribution
*Corresponding author: justin.d.krogue@gmail.com

Supplementary Material

Supplementary Methods

Embedding Models

In this work we explored three different models for generating image patch embeddings: Graph-Rise, BiT and SimCLR.

Graph-RISE

The Graph-Regularized Image Semantic Embedding (Graph-RISE) model[18] is a large scale image embedding neural network trained on approximately 40M classes from 260M web images. This model has been successfully employed in prior pathology related tasks such as image search[18,19] and generating machine-learned features survival prediction[15].

BiT

The Bit Transfer (BiT) model[20] used in the work is based on ResNet50[22] neural network architecture trained on the publicly available ImageNet[23] dataset.

SimCLR

The SimCLR[21] model used in this work was initialized with the ResNet50 BiT model described above and then trained using the SimCLR methodology on a random sample of 50M patches from 10,705 cases (29,018 slides) spanning 32 studies from The Cancer Genome Project (TCGA). This model was trained for 5M steps with a batch size of 1024 with a learning rate of 0.3 and temperature of 0.1, and was trained on V2 TPU.

Supplementary Tables

Supplementary Table S1: Cohort characteristics

The development set consists of Stages II or III cases with T-categories 3 or 4 from the Medical University of Graz from 1984 to 2007. The temporal validation set consists of Stages II or III with T-categories 3 or 4 cases from the Medical University of Graz from 2008-2013. External validation set 1b consists of Stages I-IV with T-categories 2-4 from Stanford University from 2007-2018. External validation set 1a is a subset of external validation set 1b containing only Stages II or III with T-categories 3 or 4.

Supplementary Table S2: Pathologist descriptions of machine-learned features

Feature	Description
**1**	Predominantly adipose and inflammatory cells with occasional tumor cells
**2**	Predominantly low grade adenocarcinoma and associated stroma
**3**	Predominantly moderately differentiated adenocarcinoma and occasional inflammatory and fibrotic stroma
**4**	Predominantly high grade adenocarcinoma with high tumor:stroma ratio
**5**	Predominantly low grade to moderately differentiated adenocarcinoma with occasional inflammatory cell infiltrate and intraglandular necrotic debris

Supplementary Table S3: Performance for LNM prediction using different embedding models to generate features

AUROCs for LNM predictions for logistic regressions containing baseline clinicopathologic variables (age, sex, tumor grade, T-category, lymphatic invasion, venous invasion) and the top-5 machine-learned features from different embedding models. 95% CIs computed via bootstrapping.

Dataset	Embedding Model		
	Graph-RISE	BiT	SimCLR
Temporal validation	0.715<br>[0.674, 0.753]	0.730<br>0.689, 0.766]	0.703<br>[0.660, 0.740]
External validation 1a	0.740<br>[0.701, 0.780]	0.737<br>[0.697, 0.782]	0.737<br>[0.696, 0.778]
External validation 1b	0.738<br>[0.705, 0.770]	0.731<br>[0.698, 0.763]	0.740<br>[0.706, 0.772]

Supplementary Table S4: Sensitivity, specificity, PPV, and NPV for each model using optimized threshold.

The optimized threshold was determined by selecting the value that maximized the harmonic mean of sensitivity and specificity. **Clinical**: baseline clinicopathologic variables (age, sex, tumor grade, T-category, lymphatic invasion, venous invasion). **Clinical + ML**: baseline clinicopathologic variables plus 5 machine-learned features. 95% confidence intervals computed via bootstrapping.

Model	Metric	Temporal validation	External validation 1a	External validation 1b
Clinical	Accuracy	62.4% [58.8, 65.8]	67.8% [64.0, 71.8]	67.8% [64.7, 70.7]
	Sensitivity	59.3% [54.0, 64.5]	66.3% [60.8, 71.7]	63.6% [58.8, 67.9]
	Specificity	65.8% [60.4, 70.9]	69.5% [63.6, 75.1]	71.8% [67.5, 75.4]
	PPV	66.0% [60.9, 71.1]	71.0% [65.4, 76.7]	67.4% [63.0, 71.8]
	NPV	59.1% [54.1, 64.3]	64.7% [58.9, 70.1]	68.2% [63.4, 72.2]
Clinical + ML	Accuracy	67.8% [64.0, 71.3]	68.2% [64.5, 72.2]	68.3% [65.2, 71.2]
	Sensitivity	70.1% [65.2, 74.8]	68.7% [63.5, 73.9]	65.0% [60.2, 69.4]
	Specificity	65.2% [59.6, 70.3]	67.6% [61.7, 73.0]	71.3% [67.2, 75.3]
	PPV	69.3% [64.5, 74.3]	70.4% [64.5, 75.4]	67.6% [63.3, 72.1]
	NPV	66.0% [60.6, 71.0]	65.8% [60.1, 71.6]	68.9% [64.2, 72.8]

Supplementary Table S5: AUROC for LNM prediction without accounting for baseline clinicopathologic variables during machine-learned feature selection.

AUROCs for LNM predictions for logistic regressions with various feature sets. Clinical: baseline clinicopathologic variables (age, sex, tumor grade, T-category, lymphatic invasion, venous invasion). Clinical + ML: baseline clinicopathologic variables plus 5 machine-learned features selected without controlling for baseline clinicopathologic variables. 95% confidence intervals computed via bootstrapping.

Model	Temporal validation	External validation 1a	External validation 1b
**Clinical**	0.667 [0.626, 0.708]	0.716 [0.674, 0.762]	0.719 [0.684, 0.752]
**Clinical + ML**	0.710 [0.669, 0.747]	0.706 [0.662, 0.750]	0.700 [0.666, 0.736]
**Delta**	0.042 [0.017, 0.070]	-0.010 [-0.039, 0.019]	-0.019 [-0.040, 0.002]

Supplementary Figures

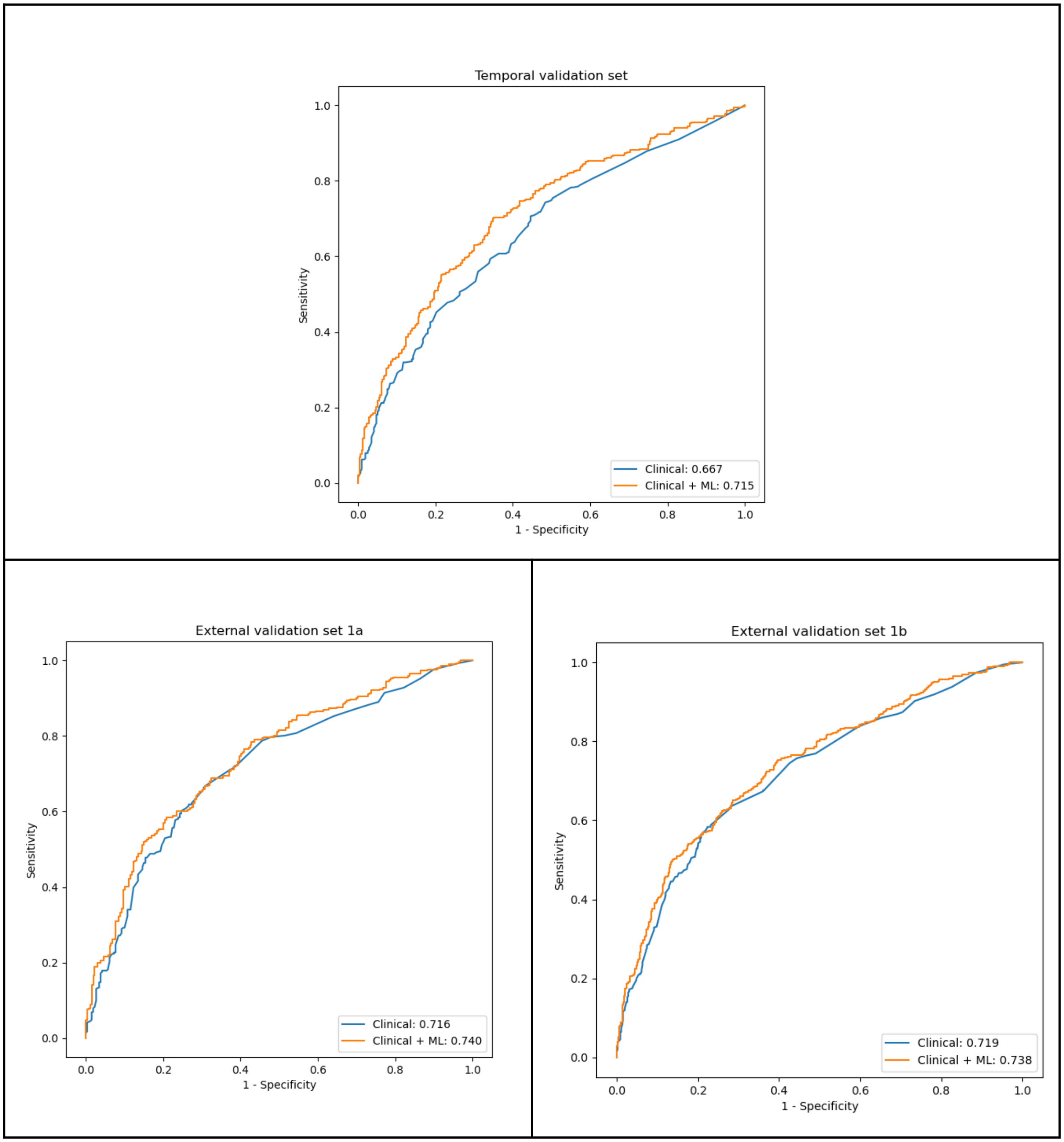

Supplementary Figure S1: Receiver operating characteristic (ROC) curves for Clinical vs Clinical + ML prediction models on validation datasets. ROCs with AUROCs for LNM predictions for logistic regressions with various feature sets. Clinical: baseline clinicopathologic variables (age, sex, tumor grade, T-category, lymphatic invasion, venous invasion). Clinical + ML: baseline clinicopathologic variables plus 5 machine-learned features.

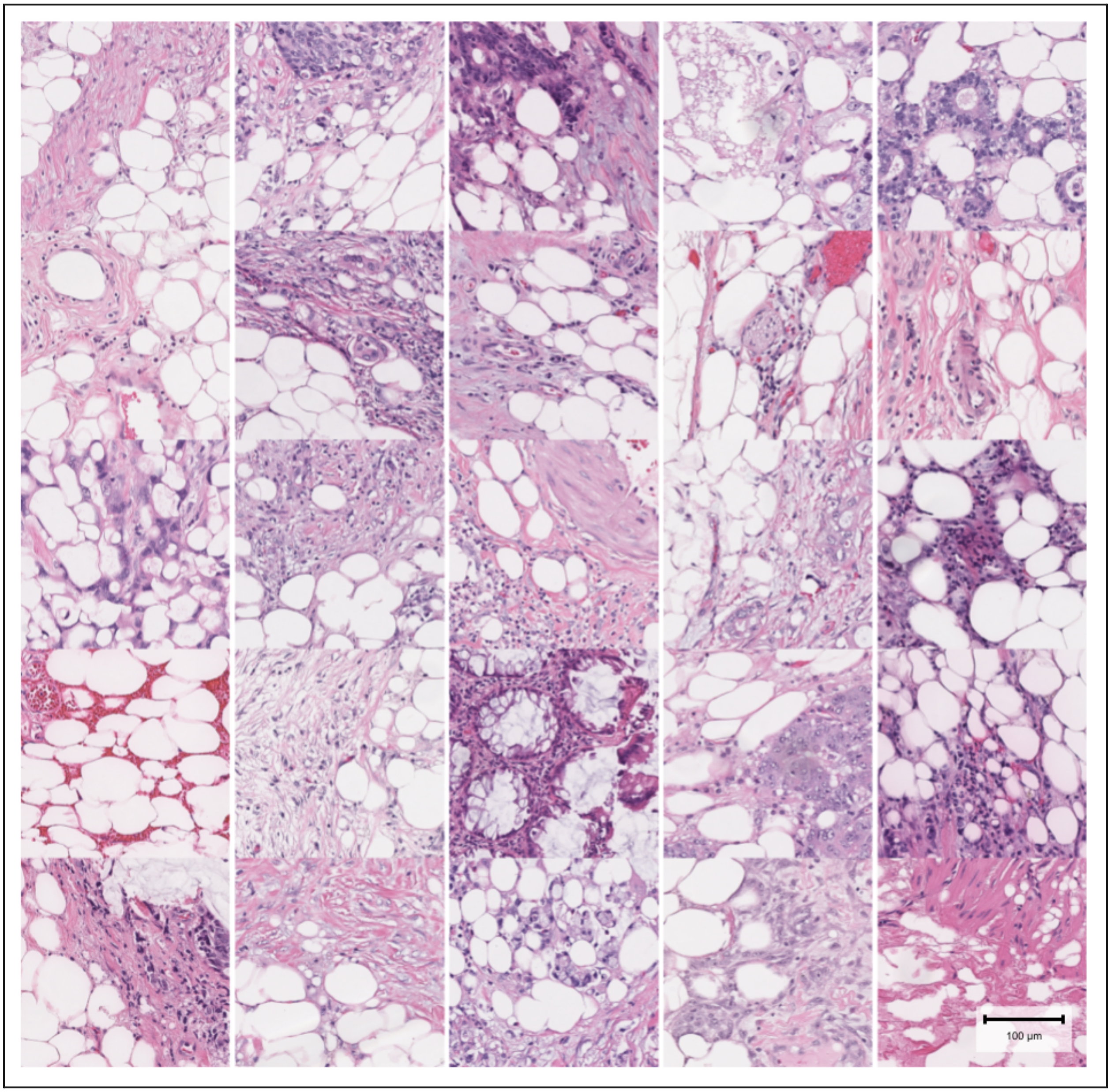

Supplementary Figure S2: Additional patches assigned to machine learning feature #1 from external validation set 1a. Patches selected here are the next 25 patches closest to the cluster centroid (after the five previously shown in Figure 2), and each patch is sampled from a unique case. Patches are 289x289 pixels obtained at 10X, with scale bar in lower right showing length of 100 micrometers.

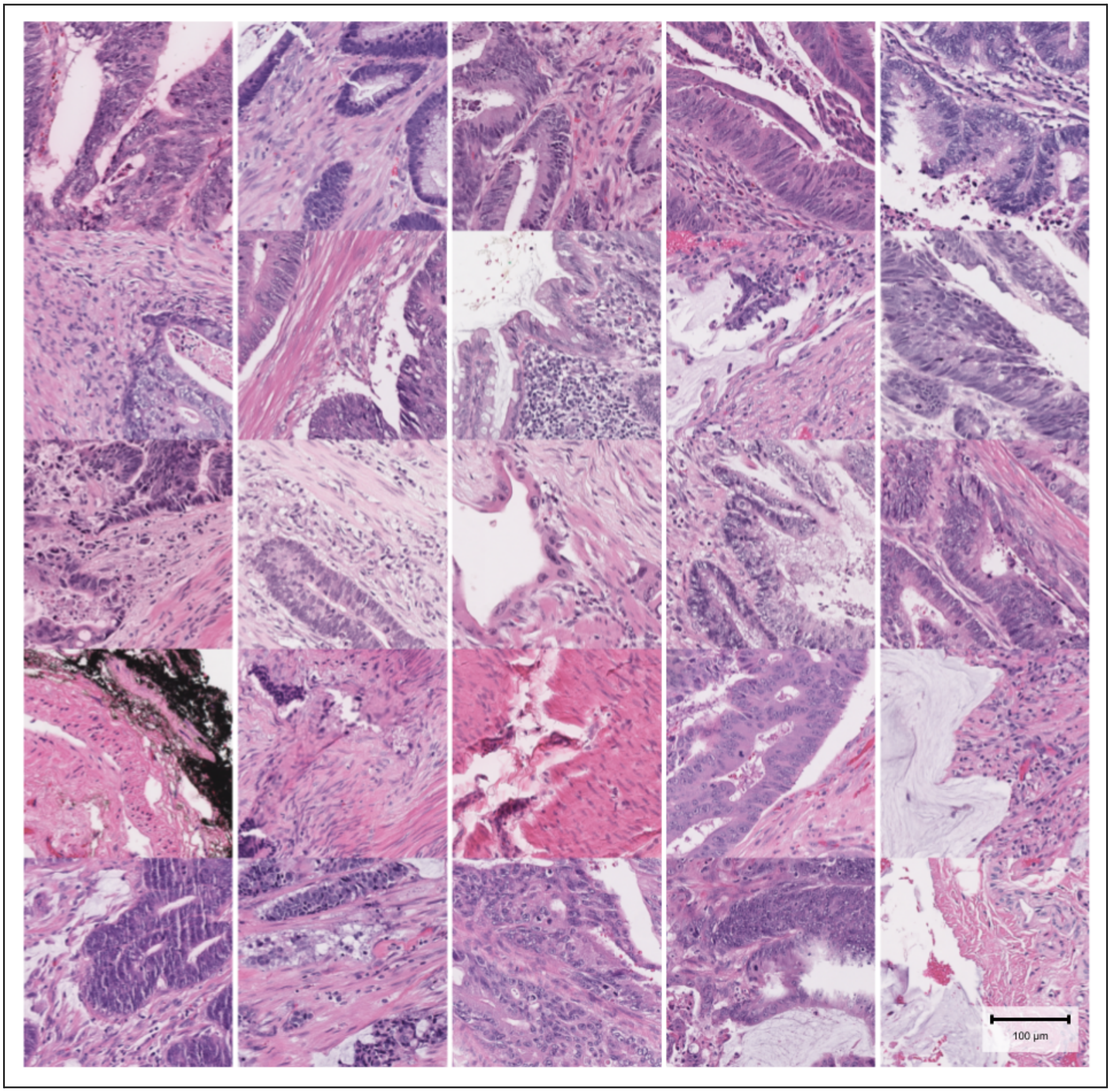

Supplementary Figure S3: Additional patches assigned to machine learning feature #2 from external validation set 1a. Patches selected here are the next 25 patches closest to the cluster centroid (after the five previously shown in Figure 2), and each patch is sampled from a unique case. Patches are 289x289 pixels obtained at 10X, with scale bar in lower right showing length of 100 micrometers.

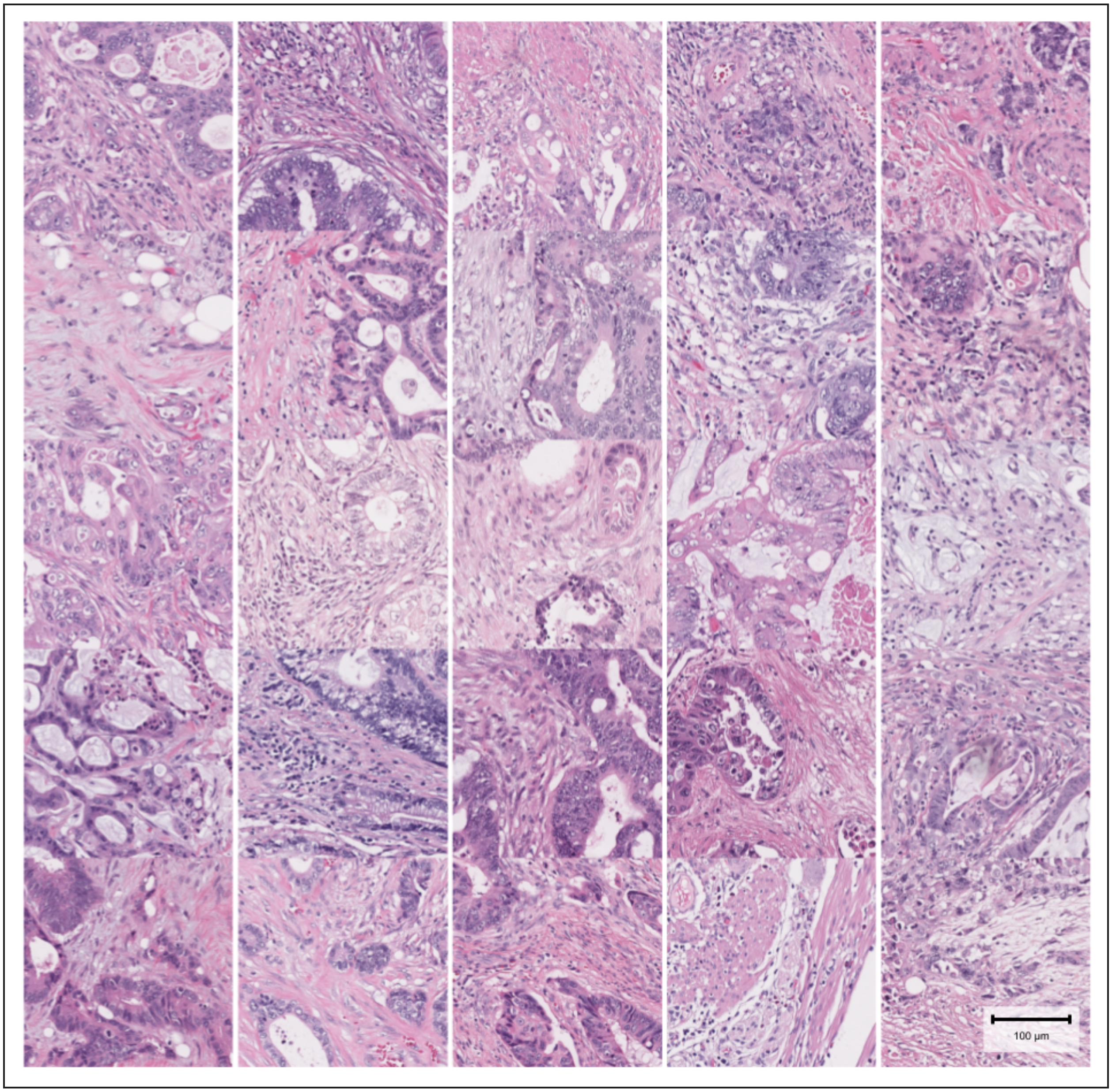

Supplementary Figure S4: Additional patches assigned to machine learning feature #3 from external validation set 1a. Patches selected here are the next 25 patches closest to the cluster centroid (after the five previously shown in Figure 2), and each patch is sampled from a unique case. Patches are 289x289 pixels obtained at 10X, with scale bar in lower right showing length of 100 micrometers.

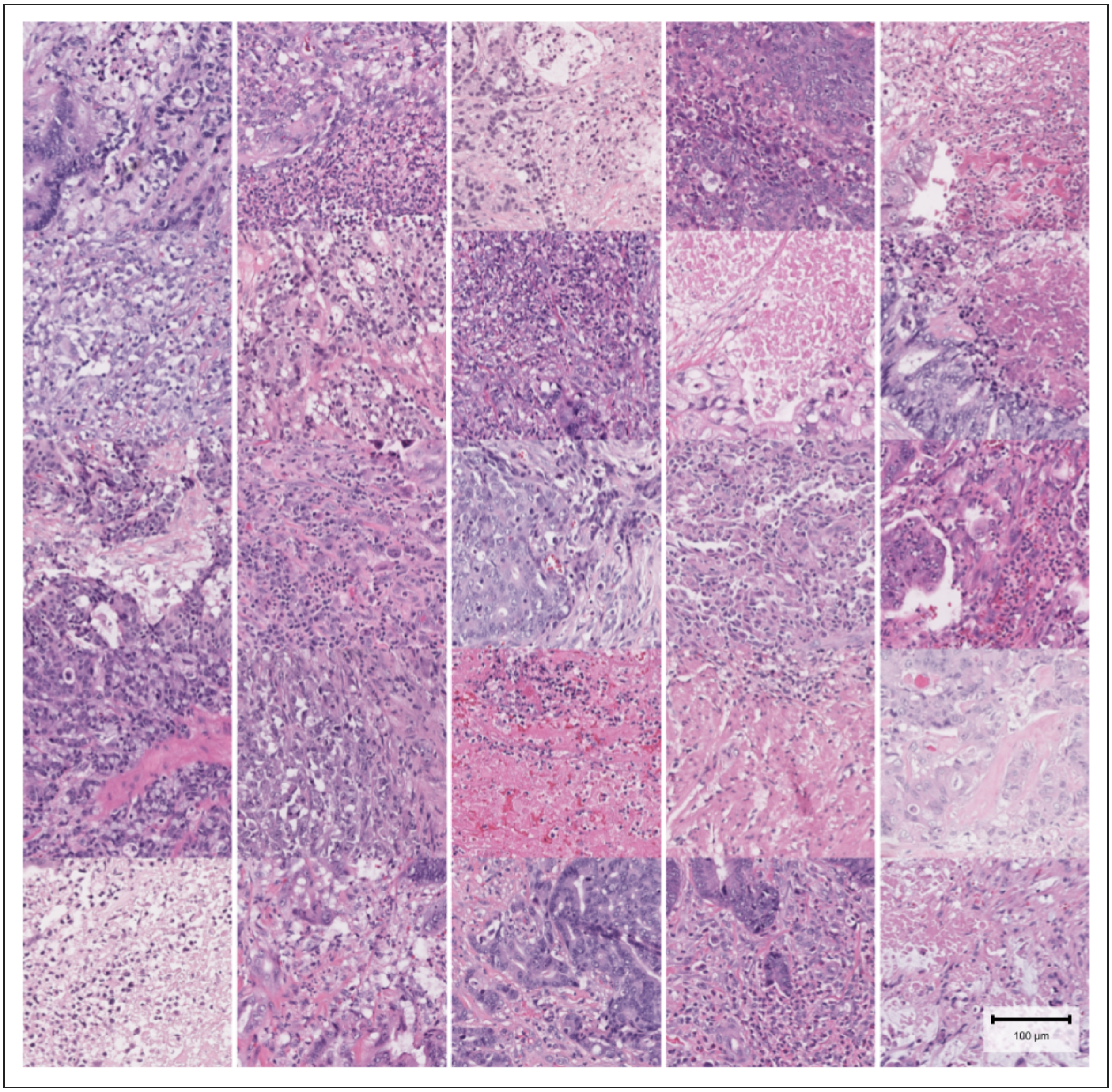

Supplementary Figure S5: Additional patches assigned to machine learning feature #4 from external validation set 1a. Patches selected here are the next 25 patches closest to the cluster centroid (after the five previously shown in Figure 2), and each patch is sampled from a unique case. Patches are 289x289 pixels obtained at 10X, with scale bar in lower right showing length of 100 micrometers.

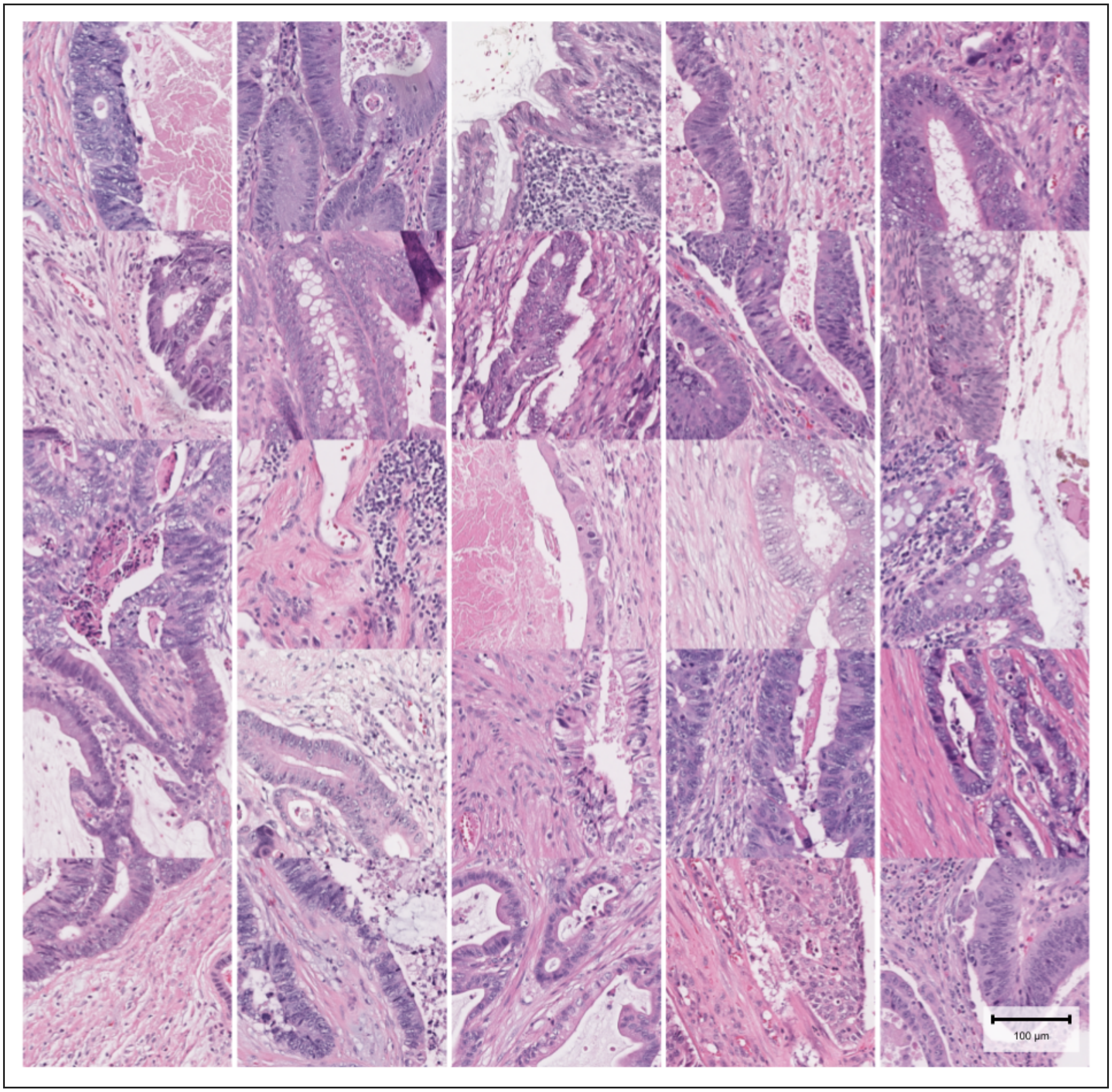

Supplementary Figure S6: Additional patches assigned to machine learning feature #5 from external validation set 1a. Patches selected here are the next 25 patches closest to the cluster centroid (after the five previously shown in Figure 2), and each patch is sampled from a unique case. Patches are 289x289 pixels obtained at 10X, with scale bar in lower right showing length of 100 micrometers.

a) BiT

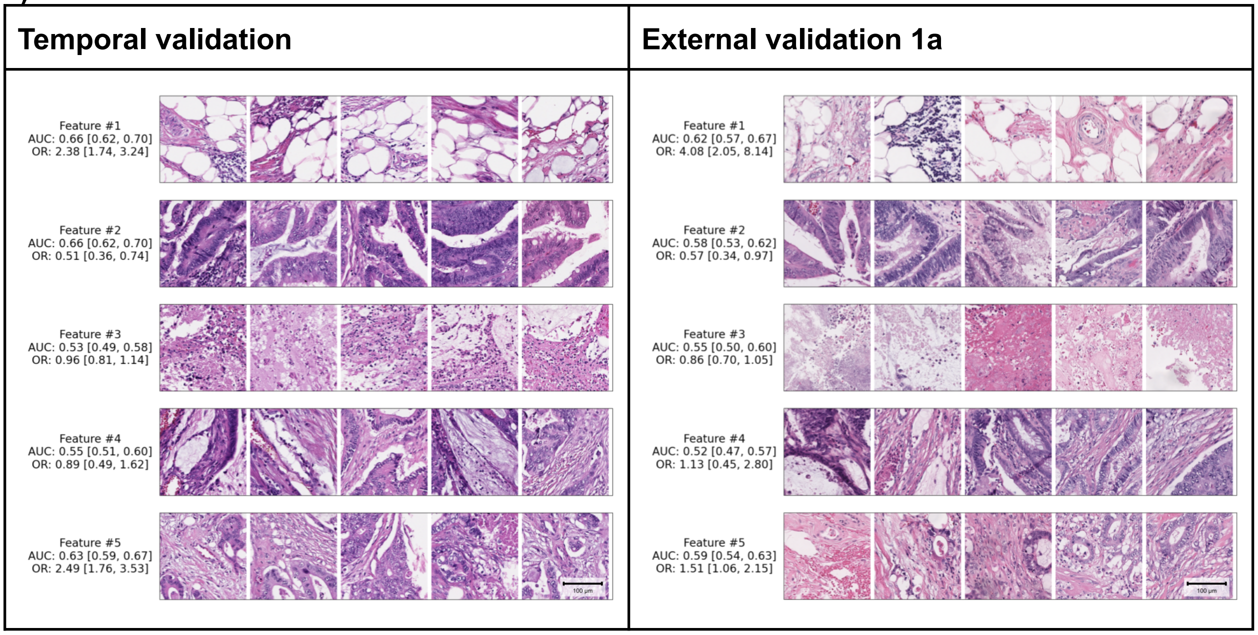

b) SimCLR

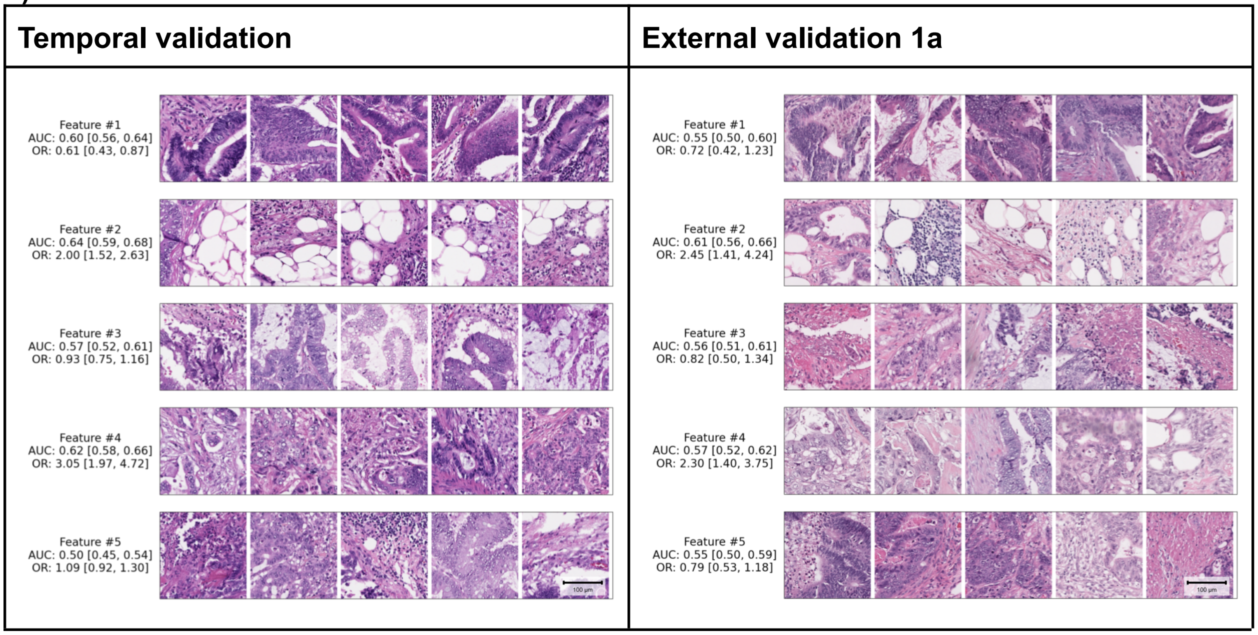

Supplementary Figure S7: Machine-learned features for alternative embedding models
Machine-learned features for temporal validation set selected using alternative embedding models: a) BiT and b) SimCLR. Patches are 224x224 pixels obtained at 10X, with scale bar in lower right showing length of 100 micrometers.

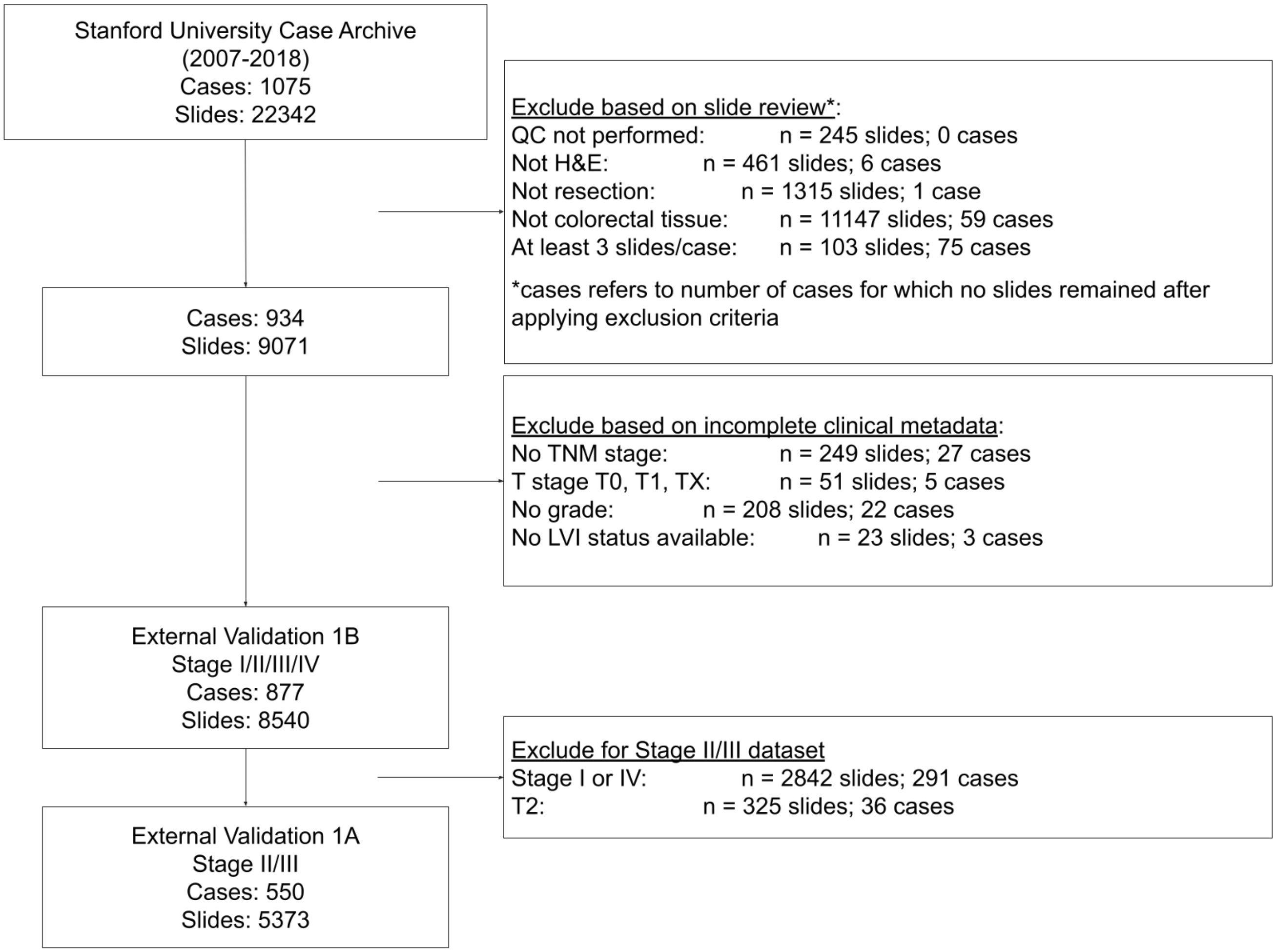

Supplementary Figure S8: STARD diagram of inclusion/exclusion criteria for external validation data cohorts.
