# Supplementary material for: Predicting lymph node metastasis from primary tumor histology and clinicopathologic factors in colorectal cancer using deep learning
Source: Commun Med (Lond). 2023 Apr 24;3:59. doi: 10.1038/s43856-023-00282-0 (PMC10125969; doi:10.1038/s43856-023-00282-0)
Supplement: Supplementary file 5 — Description of Additional Supplementary Files [file 43856_2023_282_MOESM5_ESM.pdf]

Description of Additional Supplementary Files

File Name: Supplementary Data 1

Description: Patient demographics

File Name: Supplementary Data 2

Description: Source data for Figure 3
